# Sources of Inter-individual Variability in the Therapeutic Response of Blood Glucose Control to Exercise in Type 2 Diabetes: Going Beyond Exercise Dose

**DOI:** 10.3389/fphys.2018.00896

**Published:** 2018-07-13

**Authors:** Thomas P. J. Solomon

**Affiliations:** ^1^School of Sport, Exercise, and Rehabilitation Sciences, University of Birmingham, Birmingham, United Kingdom; ^2^Institute of Metabolism and Systems Research, University of Birmingham, Birmingham, United Kingdom

**Keywords:** exercise, training, type 2 diabetes, non-responder, variability, blood glucose control, HbA1c, heterogeneity

## Abstract

In the context of type 2 diabetes, inter-individual variability in the therapeutic response of blood glucose control to exercise exists to the extent that some individuals, occasionally referred to as “non-responders,” may not experience therapeutic benefit to their blood glucose control. This narrative review examines the evidence and, more importantly, identifies the sources of such inter-individual variability. In doing so, this review highlights that no randomized controlled trial of exercise has yet prospectively measured inter-individual variability in blood glucose control in individuals with prediabetes or type 2 diabetes. Of the identified sources of inter-individual variability, neither has a prospective randomized controlled trial yet quantified the impact of exercise dose, exercise frequency, exercise type, behavioral/environmental barriers, exercise-meal timing, or anti-hyperglycemic drugs on changes in blood glucose control, in individuals with prediabetes or type 2 diabetes. In addition, there is also an urgent need for prospective trials to identify molecular or physiological predictors of inter-individual variability in the changes in blood glucose control following exercise. Therefore, the narrative identifies critical science gaps that must be filled if exercise scientists are to succeed in optimizing health care policy recommendations for type 2 diabetes, so that the therapeutic benefit of exercise may be maximized for all individuals with, or at risk of, diabetes.

## Introduction

Type 2 diabetes mellitus (T2DM) is characterized by persistent hyperglycemia (**Table 1**) that increases the risk of retinopathy, nephropathy, neuropathy, and cardiovascular-related mortality. Because diabetes affects 5–10% of the population, healthcare costs create a major socioeconomic burden. For example, in 2012 the United Kingdom, the National Health Service spent ∼10% of its annual budget (∼£24billion) on diabetes management (Hex et al., 2012). Although T2DM is considered a preventable disease, since its incidence is mostly associated with lifestyle factors, patient numbers continue to escalate.

**Table 1:** Diagnostic references range values for high risk for diabetes (prediabetes) and type 2 diabetes (American Diabetes Association, 2018a).

	Prediabetes	Type 2 diabetes
Fasting glucose	≥5.6 to 6.9 mM	≥7 mM
Two-hour OGTT glucose	≥7.8 to 11 mM	≥11.1 mM
HbA1c	≥5.7 to 6.4% (39–47 mmol/mol)	≥6.5% (48 mmol/mol)
Random blood glucose	–	≥11.1 mM


Blood glucose levels are governed by rates of glucose appearance and disappearance that are controlled by a complex interplay between metabolic, endocrine, and neurological systems. This involves direct action of neuronal, gastrointestinal, hepatic, pancreatic, renal, adipose, endothelial, and muscular tissues. Muscle contraction-mediated increases in basal glucose disposal were first documented in the 1960s (Holloszy and Narahara, 1965). In the 1970s and 1980s, it emerged that exercise also increases insulin sensitivity in rodents (Richter et al., 1982) and humans (Soman et al., 1979). An abundance of studies has now confirmed that robust increases in insulin sensitivity occur following exercise in individuals with prediabetes or patients with T2DM. Consequently, exercise is a key part of diabetes therapy included in the American Diabetes Association’s (ADA) diabetes prevention and treatment guidelines. Skeletal muscle indeed plays a large role in blood glucose uptake during/following exercise, but at rest and following a meal blood glucose levels are controlled by several tissues. Skeletal muscle insulin sensitivity is not measured in the clinic since it is impractical and because it is the exposure to persistent hyperglycemia (in additional to elevated lipids and inflammatory cytokines) that elicits diabetic complications and cardiovascular mortality. For this important reason, this review will principally focus on evidence from exercise intervention studies where blood glucose control is the primary outcome. Glycated hemoglobin (HbA1c) levels, fasting plasma glucose, and the 2-h plasma glucose value during a 75-g oral glucose tolerance test (OGTT) are the three variables used by clinicians to measure blood glucose control, and to diagnose and monitor treatment in those at risk of developing diabetes and in patients with T2DM (American Diabetes Association, 2018a) (**Table 1**).

Although there is a robust effect of exercise on insulin sensitivity, the effect of exercise training on blood glucose control is less consistent. Several reports suggest that large inter-individual variability may exist in the therapeutic effect of exercise on blood glucose control. This narrative review will explore such variability and then identify the sources of this variability. By doing so, the narrative will highlight key science gaps that must be filled in order to inform and improve the current clinical guidelines.

## Evidence for Inter-Individual Variability in the Therapeutic Effect of Exercise on Blood Glucose Control, in Individuals With (Pre)Diabetes

Clues that inter-individual variability exists have emerged from clinicians’ anecdotal observations and alarmist media headlines. However, we can venture beyond subjective assessments and objectively examine such variability. The first evidence comes from the HERITAGE family study in which Boulé and colleagues followed 596 healthy sedentary individuals through a 20-week training intervention (Boulé et al., 2005). Participants exercised on cycle ergometers for 30–50-min on 3 days/week, at 55–75% VO_2_max. Glucose tolerance and its determinants (insulin sensitivity and insulin secretion) was determined via intravenous glucose tolerance tests (IVGTT) before and after the intervention. Although there were statistically significant training-induced increases in glucose disappearance rate (K_g_), insulin sensitivity (S_i_), and disposition index (DI, a marker of insulin secretory function relative to insulin sensitivity), approximately 40% of the participants showed no change or an adverse direction of change (a decrease) in these variables (Boulé et al., 2005). Fasting glucose also significantly improved but its inter-individual variability was not reported. This work made an important advance by presenting the variability of training-induced changes in diabetes-relevant variables; however, participants with prediabetes or T2DM were not included, and neither HbA1c nor 2-h OGTT glucose were measured (Boulé et al., 2005). We followed up this work in 2013 to examine the inter-individual variability in the therapeutic response of blood glucose control in 105 older obese individuals with prediabetes or T2DM, excluding those treated with insulin (Solomon et al., 2013b). All participants underwent a 12–16-week aerobic exercise training intervention consisting of up to 60 min/day supervised walking or cycling on 4–5 days/week at up to 75% of VO_2_max. Blood glucose control (HbA1c, fasting glucose, 2-h OGTT glucose) and its determinants, insulin sensitivity (measured by hyperinsulinemic euglycemic clamp) and insulin secretion (plasma C-peptide response to OGTT), were assessed before and following the intervention. Exercise training was well adhered to and there was a small statistically significant reduction in both fasting glucose and 2-h OGTT glucose, along with an statistically significant increase in insulin-sensitivity and disposition index (Solomon et al., 2013b).

**FIGURE 1:**
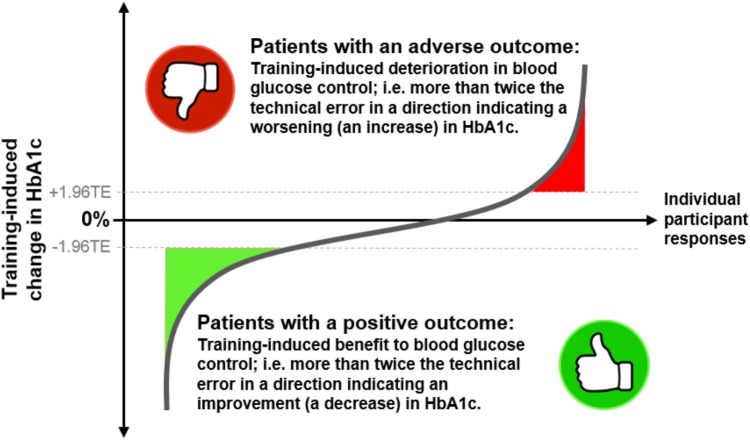
A cartoon depicting the inter-individual variability of changes in HbA1c levels following exercise training in individuals with prediabetes or type 2 diabetes. The *y*-axis represents the training-induced change in HbA1c (post- minus pre-intervention value). The *x*-axis represents the individual participants taking part in a study. Adverse outcomes are illustrated as participants’ responses showing a training-induced increase in HbA1c that is more than 1.96 times the technical error (TE). Therapeutic outcomes are shown as training-induced decreases in HbA1c that are greater than 1.96 times the technical error.

Following training, HbA1c, fasting glucose, and 2-h OGTT glucose were reduced in only 69, 62, and 68% of the study participants, respectively (Solomon et al., 2013b). This work indicated that approximately 1/3 of this cohort of individuals with prediabetes or T2DM had no improvement or even a deterioration in blood glucose control following exercise training (**Figure 1**). We confirmed that the observations made by Boulé et al. (2005) in healthy individuals are also evident in individuals with pathological blood glucose control. Similar findings were published in Álvarez et al. (2017) who examined the effect of 10-weeks of high-intensity interval training in two groups of women, 20 with normal fasting glucose and 20 with impaired fasting glucose and elevated HOMA-IR values. The authors found a statistically significantly reduction in HOMA-IR but an increase in HOMA-IR in 5 of the 20 participants (Álvarez et al., 2017). In the same year, work from Phillips and colleagues also highlighted variability in changes in HOMA-IR following training in obese or prediabetic individuals (Phillips et al., 2017). It must be highlighted, however, that neither our work (Solomon et al., 2013b) or the work of Boulé (Boulé et al., 2005), or Álvarez (Álvarez et al., 2017), included a non-exercise control group. Therefore, the direct effects of training *per se* are uncertain, and the natural variability (i.e., intra-subject variability) in the measured variables over the time course of the interventions are not known. The work from Phillips did include a non-exercise control group but did not report variability from diabetes-related clinical diagnostics (HbA1c, or blood glucose) (Phillips et al., 2017). More importantly, as will be discussed in the next section, despite concluding that inter-individual variability in the therapeutic effect of exercise on blood glucose control exists, it can be debated whether these studies employed an adequate study design in order to detect such variability and thus accurately identify adverse outcomes.

## How Should We Quantify Inter-Individual Variability and Thereby Identify a Non-Responder to Exercise?

As the previous section alludes, inter-individual training-induced changes in blood glucose control are heterogeneous across the population. The treatment goal for diabetes is to manage blood glucose control by reducing HbA1c, fasting glucose, and 2-h OGTT glucose toward specific target levels. A “non-responder” is a patient displaying a lack of therapeutic benefit (no reduction in HbA1c, in the context of this review) following treatment, while a deterioration in blood glucose control (i.e., an increase in HbA1c) is an “adverse outcome” since this confers an elevated risk of diabetic complications and cardiovascular-related mortality. Previously, this phenomenon has also been referred to as “exercise resistance” (De Filippis et al., 2008; Stephens and Sparks, 2015). Several randomized controlled trials have found no significant improvement in blood glucose control in patients with T2DM following training (Dela et al., 2004; Burns et al., 2007; Karstoft et al., 2013; Terada et al., 2013b). However, these studies present the changes in sample mean (±SD, or ±SEM) and do not provide information regarding the inter-individual responses.

To obtain an accurate assessment of the inter-individual variability in an intervention-induced change in a primary outcome variable, one must be able to quantify the two components of change: random change (induced by technical and/or biological error) and systematic change (induced by the intervention). To separate random from systematic changes, scientists may calculate the typical error of measurement (i.e., the within-subject standard deviation), which reflects the measurement-to-measurement variation in a patient’s value. Typical error is equal to the standard deviation of the sum of the observed differences between repeated measurements within each individual. Since variance is equal to standard deviation squared (s^2^), the variance of the differences between within-subject repeated-measurements (represented by s*_diff_*^2^) is equal to the sum of the variances representing the typical error. This can be written as s*_diff_*^2^ = s^2^ + s^2^ which rearranges to s = s*_diff_*/√2, therefore the technical error of measurement is equal to the standard deviation of the difference scores divided by the square root of 2. For normally distributed data, 95% of the observations fall within 1.96 standard deviations of the mean. Therefore, to have 95% confidence that an intervention (either treatment or control) has no effect on a variable of interest in a particular individual and thereby identify a non-responder, the intervention-induced change in that subject should be less than 1.96 times the technical error of measurement. In the context of this review, any diabetic patient not exhibiting an exercise-induced decrease in HbA1c (or fasting glucose or 2-h OGTT glucose) more than 1.96 times the technical error would be a true non-responder. These principles have been discussed in detail by Hopkins (2000) and Senn (2004). Technical error, however, may display heteroscedasticity. For example, it could be greater when the value of the variable is larger, or it may differ across sub-groups (e.g., male vs. female, young vs. old, diabetic vs. non-diabetic, etc.). If so, applying an average typical error to all groups may overestimate some individuals and underestimate others. To account for heteroscedasticity, the typical error of measurement would need to be calculated individually for all such sub-groups or, more simply, the data could be normalized; for example, by log transforming to remove the heteroscedasticity or by expressing the technical error as a percentage of the respective mean (i.e., a coefficient of variation for the technical error). But, how should we use technical error to quantify inter-individual variability?

**FIGURE 2:**
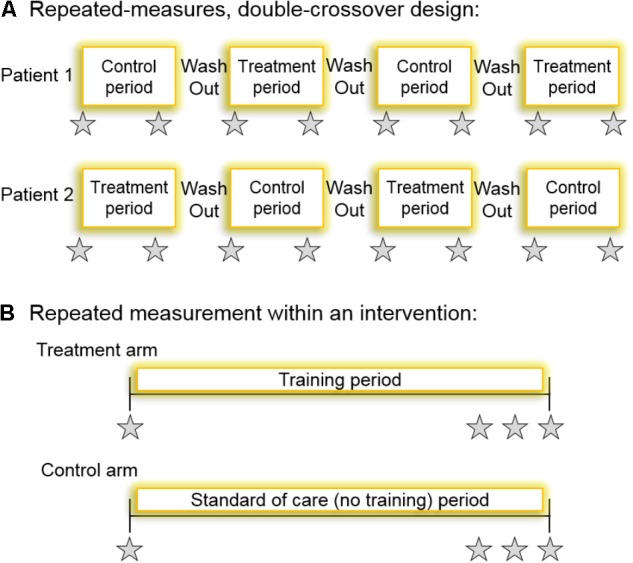
**(A)** A schematic for a repeated-measures double-crossover study design which, if the patient-by-treatment interaction term were statistically significant, would accurately indicate the presence of inter-individual variability. All patients undergo all interventions twice. The type of trial each participant first undergoes should be randomized and the primary outcome is measured at the beginning and the end of each intervention period. The time between interventions would have to be sufficient such that training effect was washed out. **(B)** Perhaps a more practical and logistically feasible method for determining the technical error of measurement, and thereby identifying non-responders, is a randomized controlled intervention where the primary outcome variable is measured repeatedly within an intervention. Participants are randomized to either the control arm (no training) or the treatment arm (exercise training). Ideally, the variable would be repeatedly measured within a time frame where intervention-induced changes are unlikely (e.g., measuring HbA1c three times within a 2-week period towards the end of a long-term training intervention). Gray stars indicate repeated measurement of the variable, HbA1c, for example.

In a repeated-measures study, if data are analyzed using a linear mixed model with the patient ID number as a random effect and the intervention assigned as a fixed effect, a significant patient-by-treatment interaction would indicate true inter-individual variability. However, this would only be correct if the training effect on an individual is reproducible. Naturally, the blood glucose lowering response to exercise training in T2DM may not only exhibit inter-individual variability but also intra-individual variability of the measurement if the intervention was repeated within an individual. Consequently, the optimal approach for quantifying inter-individual variability in repeated-measures studies is to use a randomized replicated crossover design where the control (no training) and treatment (training) conditions are administered to each participant at least twice (Senn et al., 2011; Hecksteden et al., 2015; Atkinson et al., 2018; Goltz et al., 2018) (**Figure 2**). This design would allow accurate interpretation of a significant patient-by-treatment interaction, thereby revealing the true individual differences in response to exercise. A limitation of this approach, however, is that an adequate wash-out of the training effects would be required which, for exercise studies, creates logistical difficulty. It is unlikely that long-term training studies with a double crossover to determine the patient-by-treatment interaction effect on blood glucose control will ever be conducted in patients with T2DM (**Figure 2**), because blood glucose control would deteriorate while patients were not exercising, and several confounding variables would likely change (e.g., their drug regimen, body weight, etc.). Hecksteden et al. (2015) proposed an indirect approach whereby a separate validity study would be conducted to determine the within-subject variability in their response to repeated training interventions and using a linear mixed model to apply that to the main study. Although not in the context of blood glucose control or in individuals with (pre)diabetes, some training studies have used this approach (Bonafiglia et al., 2016; Gurd et al., 2016) but it is confounded by assumed generalizability, which contradicts the initial reason to accurately determine whether true inter-individual variability actually exists. Fortunately, a more practical alternative solution exists which is to repeatedly test the primary outcome variable within an intervention. Bouchard et al. (2012) explored inter-individual variability in the response of metabolic syndrome related variables to exercise training in order to identify adverse outcomes. They used an approach whereby resting systolic blood pressure, fasting triglycerides, and fasting HDL-cholesterol were measured three times over a 3-week period in sixty subjects from six independent randomized controlled long-term exercise training studies (including HERITAGE, DREW, INFLAME, STRRIDE, and others). Subsequently, they calculated the technical error of measurement for these variables to determine the frequency of exercise-induced adverse outcomes, reporting that 12, 10, and 13% of their sample population had an “adverse response” in systolic blood pressure, triglycerides, and HDL-cholesterol, respectively, following exercise training (Bouchard et al., 2012). While this elegant approach, which was also used by Phillips et al. (2017), provides evidence that non-responders to exercise indeed exist in the context of cardiometabolic risk factors, surprisingly the authors of neither study presented inter-individual changes in blood glucose control. Blood glucose control (2-h OGTT glucose) was measured, however, by De Lannoy et al. (2017) who found that the number of non-responders ranged from 86 to 98% following different types of training in 171 obese non-diabetic adults.

The above-mentioned studies provide evidence for inter-individual variability in the therapeutic effect of exercise on blood glucose control, in individuals with (pre)diabetes. However, as of 2018, there is an urgent need for a large-scale randomized controlled trial aimed specifically at investigating the variability of long-term training adaptations in blood glucose control in individuals with T2DM. Such a trial should employ a study design allowing analysis of a patient-by-treatment interaction. This would be possible with a repeated-measures crossover where the control and treatment conditions are administered to each participant at least twice, or where the primary outcome variable (HbA1c, fasting glucose, and 2-h OGTT glucose) is repeatedly tested within the control and treatment intervention arms (**Figure 2**). As described above, both approaches have their limitations that investigators need to be aware of. But such approaches would generate technical errors of measurement, enabling training-induced effects on blood glucose control to be reliably compared between independent studies, and allowing interpretations to be made in the context of clinically meaningful responses to interventions in individual subjects. The eternal endeavor of achieving statistical significance between means is useless when trying to identify whether one particular person has responded or not to a treatment. Therefore, measuring technical error, quantifying inter-individual variability, and thereby detecting true adverse outcomes in exercise science will advance the field. However, this will only be achieved if investigators also attempt to control for as many sources of variance as possible.

## Sources of Inter-Individual Variability in the Therapeutic Effect of Exercise on Blood Glucose Control in Individuals With (Pre)Diabetes

The above-described evidence suggests it is very likely that inter-individual variability in the therapeutic effects of exercise on blood glucose control truly exists in patients with T2DM, and that adverse outcomes do occur. However, we do not precisely understand what causes adverse outcomes and, more importantly therefore, we do not currently know how adverse outcomes can be prevented. The standard-of-care guidelines for diabetes which are published annually by the American Diabetes Association, provide excellent, clear and effective evidence-based exercise recommendations that are summarized in **Table 2** (American Diabetes Association, 2018b). However, heterogeneity in the therapeutic effect of exercise between patients inevitably prompts us to explore how adverse outcomes can be avoided. Exercise scientists, clinicians, and fitness trainers often comment that non-responders should simply do more exercise. It is indeed enticing to believe such a sentiment, particularly when it may be true in the context of improving cardiorespiratory fitness (VO_2_max) in healthy adults (Montero and Lundby, 2017). Nonetheless, in individuals with (pre)diabetes, several lines of published evidence have identified sources of inter-individual variability in the therapeutic effect of exercise on blood glucose that go beyond exercise dose. The following points discuss these sources in the context of current guidelines which, with future experimental evidence, will be improved by providing more information on exercise dose, exercise type, exercise-meal timing, and anti-hyperglycemic drug-exercise timing, etc.

**Table 2:** Exercise recommendations for adults with prediabetes or type 2 diabetes, issued in American Diabetes Association (2018b) standard of care update.

Exercise recommendations for adults with prediabetes or type 2 diabetes	
1	≥150 min of moderate to vigorous intensity aerobic activity per week, spread over at least 3 days/week, with no more than 2 consecutive days without activity.
2	Shorter duration (≥75 min per week) of vigorous-intensity or interval training may be sufficient for younger and more physically fit individuals.
3	Two to three sessions per week of resistance exercise on non-consecutive days.
4	Decrease the amount of time spent in daily sedentary behavior. Prolonged sitting should be interrupted every 30 min.
5	Flexibility training and balance training are recommended 2–3 times per week for older adults with diabetes.


### A. Exercise Dose (Frequency, Intensity, and Time)

The first evidence that exercise dose might contribute to the inter-individual variability of training-induced changes in blood glucose control came from the elegant series of STRRIDE studies. In 2004, over 200 middle-older aged, sedentary, overweight or obese individuals were randomized to one of four 8-month training interventions: (i) low amount/moderate intensity (1200 kcal/week), (ii) low amount/vigorous intensity (1200 kcal/week), (iii) high amount/vigorous intensity (2000 kcal/week), or (iv) non-exercise control. Fasting glucose, and insulin sensitivity, insulin secretion, and disposition index (modeled from an IVGTT), were measured before and after the interventions. Fasting glucose increased in the control group but was unaffected by any of the training interventions, whereas insulin sensitivity was most increased by the low amount/moderate intensity and high amount/vigorous intensity interventions compared to the low amount/vigorous intensity intervention (Houmard et al., 2004; Slentz et al., 2009). Of importance, the expected training-induced compensatory decrease in the insulin secretory response to IVGTT (relative to the increased insulin sensitivity) was smallest following the low amount/moderate intensity intervention, leading to the largest increase in glucose disposition in that group (Slentz et al., 2009). The STRRIDE investigators also followed up these investigations in ∼150 middle- to older-aged, sedentary participants with prediabetes randomized to one of four 6-month interventions: (i) low amount/moderate intensity exercise, (ii) high amount/moderate-intensity exercise, (iii) high amount/vigorous-intensity exercise, or (iv) control group (low amount/moderate intensity plus 7% weight loss to mimic the DPP study). This STRRIDE-Prediabetes study (Slentz et al., 2016) found that only the control group improved fasting glucose but that high amount/moderate-intensity exercise was most effective for lowering the blood glucose during OGTT, when compared to either low amount/moderate intensity or high amount/vigorous-intensity exercise. A further study (Malin et al., 2013) found that exercise dose was positively associated with increased glucose disposition index following a 3-month aerobic training intervention in 35 older, obese, individuals with prediabetes. However, no statistically significant associations between exercise dose and changes in blood glucose control were found (Malin et al., 2013). Furthermore, exercise dose was simply estimated from assumed energy expenditure during exercise sessions in a supervised training intervention and participants were not randomized to different exercise dosing groups (Malin et al., 2013). Finally, Terada and colleagues conducted a retrospective analysis of outcomes in 15 middle- to older-aged individuals with T2DM randomly assigned to a 12-week energy expenditure-matched high-intensity interval exercise or moderate-intensity steady-state exercise intervention (Terada et al., 2013a). Capillary blood glucose was measured immediately before and after each exercise bout and multiple regression analyses demonstrated that greater reductions in blood glucose were found in individuals working at higher exercise intensities or engaged in longer exercise bouts (45- or 60- vs. 30-min). Hence, Terada et al. (2013a) showed that a larger exercise dose was more effective for reducing hyperglycemia in diabetes patients.

Exercise frequency has seldom been studied during training interventions in the context of diabetes. Dubé et al. (2012), who studied the effects of 16-weeks of aerobic exercise training on changes in insulin sensitivity in middle-aged overweight and obese non-diabetic individuals, found that although exercise dose was positively correlated with increased insulin sensitivity, exercise frequency did not contribute to the magnitude of the change. Unfortunately, Dubé and colleagues did not report blood glucose control variables (Dubé et al., 2012). Exercise frequency has, however, been studied in detail in people with (pre)diabetes during a 24–48-h period. In van Dijk et al. (2012) found that a 30-min cycle at 50% peak power on two consecutive days elicited the same improvement in continuous glucose monitoring (CGM)-derived blood glucose control compared to a single 60-min ride in thirty older obese patients with T2DM. DiPietro et al. (2013) found that three 15-min post-meal walking bouts (moderate-intensity; 3 METs) was more effective at lowering CGM-derived blood glucose profiles than a single 45-min bout performed in the morning or evening, in older adults with prediabetes. In a similar demographic of prediabetic adults, in Francois et al. (2014) also found that walking bouts (6 × 1 min incline walking at 90% HRmax) performed 30 min before the three meals of the day reduced CGM-derived glucose compared to no exercise or a single 30-min bout of moderate-intensity (60% of maximal heart rate) walking. However, these studies (DiPietro et al., 2013; Francois et al., 2014), along with the work of Dubé et al. (2012) did not present the inter-individual responses. While some studies indicate that exercise dose may indeed influence variability in the changes in blood glucose control following training in people with (pre)diabetes, no study has yet specifically analyzed the inter-individual variability caused by different exercise doses or frequency. While current guidelines (**Table 2**) for preventing and treating T2DM clearly state how many minutes of exercise should be accumulated each week and how frequent exercise sessions should be (American Diabetes Association, 2018b), precise guidance on what a moderate to vigorous intensity equates to is lacking.

### B. Exercise Type

Evidence that the type of exercise might play a role in the inter-individual variability in outcomes also originates from the STRIDE team. In 2011, the STRRIDE-AT/RT study randomized ∼200 volunteers to (i) resistance training (3 days/week, 3 sets/day of 8–12 repetitions of 8 different exercises targeting all major muscle groups), (ii) aerobic training (∼120 min/week at 75% of the VO_2_max), or (iii) combined resistance plus aerobic training, for 8-months (Bateman et al., 2011). Although the post-minus pre-intervention change in blood glucose increased in the combined resistance-aerobic group and decreased in the aerobic-only and resistance-only group, the large standard error of the change scores indicates probable heterogeneity between subjects. Consequently, the authors found no statistically significant effects of exercise on fasting blood glucose nor statistical differences between groups (Bateman et al., 2011). The HART-D and DARE studies advanced this work by conducting randomized controlled trials comparing the effects of resistance, aerobic, vs. combined resistance-aerobic training on HbA1c in patients with T2DM (Sigal et al., 2007; Church et al., 2010). Both studies employed similar designs, training sessions were fully supervised, but HART-D was longer in duration than DARE (9- vs. 6-months) and made more thorough recording and monitoring of the exercise dose and energy expenditure. HART-D found that only combined resistance-aerobic training significantly reduced HbA1c (Church et al., 2010), while HbA1c was significantly reduced in all 3 exercise groups the DARE trial (Sigal et al., 2007). The between-study differences may be attributable to the greater weight loss (fat mass) seen in DARE vs. HART-D. Further to such work, a randomized controlled trial from my group compared the effects of 4-months of moderate-intensity steady-state walking training (4–5 days/week, ∼60 min/day) vs. energy expenditure matched moderate-intensity interval walking training (ten cycles of 3-min fast, 3- min slow walking) in patients with T2DM (Karstoft et al., 2013). We found that only interval walking training improved CGM-derived glucose control but that this was in the presence of greater weight loss than the continuous walking group. We also found that varying interval length (1-min fast, 1 min slow vs. 3-min fast, 3- min slow walking) had no influence on the improvement in blood glucose control (Jakobsen et al., 2016).

The current guidelines (**Table 2**) do not provide specific guidance on which types of exercise may be used (American Diabetes Association, 2018b). Neither are they explicit on what aerobic or resistance exercise means. Some studies indeed indicate that either aerobic or resistance exercise alone may be sufficient to improve blood glucose control, while other work shows that different modalities of walking can also have diverging outcomes. In an age of interval training popularity, since 2017 the ADA guidelines have included a useful statement concerning vigorous exercise and interval training (American Diabetes Association, 2018b), stating that “≥75 min/week of vigorous-intensity or interval training may be sufficient for younger and more physically fit individuals.” However, specific details on what vigorous-intensity or interval exercise may entail is lacking. We also await outcomes from large scale randomized controlled trials with HbA1c as a primary endpoint to further understand the role of exercise type (particularly high intensity interval training) in order to further optimize existing guidelines. Additionally, no current study has yet prospectively examined inter-individual variability in changes in blood glucose control following different types of training in patients with prediabetes or T2DM.

### C. Exercise Adherence

In this context, adherence refers to the longevity of maintaining regular exercise following the initial inclusion of exercise to an individual’s lifestyle. With the exception of a few studies (Houmard et al., 2004; Sigal et al., 2007; Slentz et al., 2009; Church et al., 2010; Karstoft et al., 2013; Solomon et al., 2013b), exercise adherence is seldom measured or reported in training studies whose primary outcome was blood glucose lowering in (pre)diabetes patients. This is unfortunate since adherence is of utmost importance for assessing the intended stimulus provided by the intervention. That said, many trials exclude patients participating in <80% of the treatment intervention. This creates widespread homogeneity and bias. Nonetheless, a lack of adherence will clearly influence the total exercise dose (daily dose, weekly frequency), which as described above contributes toward inter-individual outcomes. However, exercise adherence goes beyond metabolism and involves health psychology (desire, motivation), behavioral barriers (e.g., self-esteem, self-image), and environmental barriers (e.g., local access to trails/parks/gyms, weather, climate). Such factors are beyond the scope of this review but have been reviewed elsewhere (Wing et al., 2001). Nonetheless, in the interest of implementing and sustaining population-wide physical activity lifestyles changes, a brief description of the influence of such factors on exercise adherence should be included in the current ADA guidelines. This may help clinicians provide more individualized advice. Furthermore, the role of adherence in the inter-individual variability in glycemic outcomes following training in (pre)diabetes patients must be studied in greater depth. This would help inform and further enhance the guidelines.

### D. Exercise-Meal Timing

Training in the fed vs. fasted state has long been an intense area of investigation for optimizing athletes’ training methods to maximize their performance. This is also true for patients with type 1 diabetes, where several studies have investigated exercise timing in relation to insulin dosing and carbohydrate intake. However, in the context of T2DM, little data exists. In the early 2000s, a series of elegant studies by Poirier and colleagues (Poirier et al., 2000, 2001; Gaudet-Savard et al., 2007) examined fed vs. fasted exercise in men with T2DM. One-hour of moderate-intensity cycling (60% VO_2_max) completed 2-h after breakfast reduced blood glucose, whereas fasted exercise did not (Poirier et al., 2001). Further, following a 3-month training intervention (1-h of cycling at 60% VO_2_max, three times per week) no change in blood glucose was found when exercise was performed in the fasted state, whereas 20–40% decreases in blood glucose arose when exercise was initiated postprandially (Poirier et al., 2000). However, the authors also documented that the effect of exercise on blood glucose in the fasted state was dependent on the ambient glucose level: blood glucose increased when pre-exercise glucose levels were ≤6 mM but decreased when pre-exercise levels were >8 mM (Gaudet-Savard et al., 2007). In support of these findings, Colberg et al. (2009) found that 20 min of self-paced treadmill walking 15–20 min after eating dinner lowered blood glucose in patients with T2DM, whereas pre-dinner exercise had no effect on blood glucose levels. Data somewhat related to these observations were generated from a retrospective analysis of outcomes from a 12-week training study in patients with T2DM conducted by Terada et al. (2013a). They demonstrated that greater reductions in blood glucose were found when pre-exercise meals were ingested less than 2-h prior to the beginning of exercise sessions rather than more than 2-h. These above-described data led to a series of view-point papers published by Chacko (2014, 2016) who presented an idea that the mid-postprandial period (30–120 min post-ingestion) would be the best time to implement exercise in order to optimize blood glucose control. While Chacko’s viewpoints are mostly anecdotal and informed by educated opinion rather than evidence, they nicely highlighted the necessity for research data in this field from a clinician’s perspective. That said, Chacko’s points are specific to the acute response to exercise. In healthy individuals, ingestion of carbohydrate prior to exercise has been shown to blunt adaptations to training (Van Proeyen et al., 2010) as well as acute bouts of exercise (Gonzalez et al., 2013; Taylor et al., 2018). Thus optimal benefit may be conferred from fasted exercise. However, in people with diabetes, fasted exercise promotes post-exercise hypoglycemia which should be avoided at all costs.

Exercise-meal timing is rarely considered in the design of training studies. As of 2018, no long-term, randomized, controlled, physical activity or exercise intervention trial with a primary focus on blood glucose control has reported exercise-meal timing. It is therefore possible that inappropriate exercise-meal timing partly explains the lack of improvement in blood glucose control in some long-term training studies in individuals with (pre)diabetes (Dela et al., 2004; Burns et al., 2007; Karstoft et al., 2013; Terada et al., 2013b). Despite efforts to elucidate the optimal exercise-meal timing, full knowledge in this area is lacking. Consequently, as of 2018, no information regarding exercise-meal timing is provided in the ADA guidelines for preventing and treating T2DM (American Diabetes Association, 2018b). Furthermore, no study has yet determined the influence of exercise-meal timing on inter-individual variability in glycemic outcomes. Given recent knowledge that skeletal muscle metabolism in humans follows a diurnal pattern under the control of clock genes (Loizides-Mangold et al., 2017), it is plausible that circadian rhythm is an additional (albeit complicated) factor to additionally consider in future work aimed at optimizing exercise-meal timing for maximal postprandial glucose control.

### E. Exercise-Drug Interactions

Anti-hyperglycemic pharmacologic therapy is administered in conjunction with lifestyle management for the treatment of, and to an extent the prevention of, T2DM. At first, metformin monotherapy is initiated to lower hepatic glucose output. If this is not successful at achieving HbA1c targets, dual or triple therapy with various insulin sensitizers (e.g., TZDs like pioglitazone), insulin secretagogues (e.g., sulfonylureas like glimepiride, or GLP-1 receptor agonists like liraglutide), DPPIV inhibitors (e.g., sitagliptin), or sodium-glucose cotransport inhibitors (e.g., canagliflozin) is initiated, with additional insulin injection therapy if HbA1c targets are still not achieved (American Diabetes Association, 2018c). Because pharmacologic therapy is always administered in conjunction with lifestyle management (diet and exercise), it is highly likely that a patient with T2DM who initiates exercise will also be using some anti-hyperglycemic medication. Since exercise affects most of the molecular pathways these compounds target, it is a clinical necessity that researchers understand exercise-drug interactions. Fortunately, several studies have examined this topic.

Sharoff et al. (2010) found that metformin (2000 mg twice/day for 2–3 weeks) did not augment the insulin sensitizing effect of exercise (40-min cycling at 65% VO_2_peak) in non-diabetic individuals and may even blunt the beneficial effects of exercise. They followed up by examining the effects of 12-weeks of exercise training (45-min of cycling at 75% HRmax 3-times/week and 2-sets of 12-rep max lifts for all major muscle groups 2-times/week) ± metformin treatment (2000 mg/day) in prediabetic individuals (Malin et al., 2012). They found that the largest increase in insulin sensitivity was present in the exercise only group, compared to the exercise plus metformin and metformin-only groups (Malin et al., 2012). Neither study found an effect on fasting glucose, while HbA1c and OGTT data were not reported. The notion that an interaction between exercise and metformin may blunt therapeutic benefits was supported by a small study in patients with T2DM from Boulé et al. (2011) which showed that exercise reduced the metformin-induced lowering of blood glucose responses to a meal. However, a larger-scale but retrospective analyses of the DARE trial in patients with T2DM from the same authors (Boulé et al., 2013) showed that improvements in HbA1c following 22-weeks of either aerobic, resistance, or combined aerobic plus resistance training, were not different between metformin users (*N* = 143) and non-users (*N* = 82). Erickson et al. (2017a) examined T2DM patients treated with either metformin monotherapy or metformin combined with additional antidiabetic drugs (sulfonylureas, GLP-1 receptor agonists, or DPPiv inhibitors) (Erickson et al., 2017b). They found that post-meal treadmill walking (five 10-min bouts at 60% VO_2_max, or three 10-min bouts at 50% VO_2_max) reduced postprandial glucose responses in habitual metformin users but that benefits were blunted in those on additional therapy (Erickson et al., 2017a,b). Such studies support the use exercise for managing blood glucose in drug-treated diabetes patients, but had a very small sample size (*N* = 8–10) and were not specifically designed to test exercise-drug interactions.

Besides metformin, several groups have examined exercise-drug interactions for other anti-diabetic drugs. In a longitudinal study by Mensberg et al. (2014), 33 T2DM patients were randomly allocated to 16-weeks of exercise (combined aerobic and resistance) and the GLP-1 receptor agonist, liraglutide (1.8 mg/day), or exercise and placebo (Mensberg et al., 2014). The authors found that HbA1c and fasting glucose were more greatly reduced in the liraglutide-treated group, indicating a beneficial interaction between exercise and GLP-1 receptor agonist therapy for blood glucose management (Mensberg et al., 2014). However, the liraglutide-treated patients also lost more weight, negating the specificity of an exercise-liraglutide interaction directly optimizing glucose control (Mensberg et al., 2014). With a focus on skeletal muscle, using fluorine-18–labeled fluoro-deoxy-glucose and positron emission tomography (PET), Hällsten and colleagues studied the effects of 26-weeks of rosiglitazone (4 mg twice daily) or metformin (1000 mg twice daily) treatment in 45 newly diagnosed patients with T2DM (Hällsten et al., 2002). Despite equal improvements in HbA1c, insulin stimulated muscle glucose uptake and exercise-induced glucose uptake (65-min of single-leg knee-extension at 10% of maximal isometric force) was augmented in the rosiglitazone group but not in the metformin-treated patients (Hällsten et al., 2002). These findings are, however, in keeping with the mechanisms of actions of these drugs. Besides insulin sensitizers, insulin secretagogues have also been studied. Larsen et al. (1999) found that the blood glucose lowering actions of the sulfonylurea glibenclamide (7 mg) and exercise (60-min at 57% of VO_2_max) are additive in patients with T2DM. Similarly, Massi-Benedetti et al. (1996) studied the glucose and insulin responses to a single exercise bout in 167 patients with T2DM treated for 14–28 days with glimepiride (3 mg/day) or glibenclamide (10 mg/day). They found that 1-h of cycling at 120 bpm reduced blood glucose in both groups, but lowered endogenous insulin secretion (as shown by reduced C-peptide levels) in the glimepiride group only (Massi-Benedetti et al., 1996). This likely indicates that glimepiride, but not glibenclamide, treatment also increases insulin- and/or- exercise-induced glucose uptake. This idea is supported by *in vitro* observations from Haupt and colleagues who showed that glimepiride activates PI3 kinase and increases insulin-stimulated glycogen synthesis in human primary skeletal muscle cells, where glibenclamide has no effect (Haupt et al., 2002).

While the majority of T2DM patients will use metformin and other anti-hyperglycemic drugs, insulin is used as a final approach and has been seldom studied in such patients in the context of exercise. van Dijk et al. (2012) found that 24-h CGM-derived hyperglycemia (time above 7.8 mM) and glycemic variability was reduced in sixty patients who had completed a 45–60-min cycling bout at 30–50%Wmax, and that this improvement was not different between insulin-treated or insulin-naïve patients (Van dijk et al., 2013). The same study also reported inter-individual differences in changes in mean 24-h glucose, finding poorer glycemic variability in nine (15%) of the sixty patients (Van dijk et al., 2013). Furthermore, while the study found that the prevalence of hypoglycemia was greater in insulin-treated patients compared with non-insulin-treated patients, this was not influenced by exercise. While hypoglycemia is a common fear and consequence of the use of exercise in insulin-treated diabetes patients, the risk of hypoglycemia can be dramatically reduced through proper instruction, advice, and guidance on carbohydrate intake and insulin-meal-exercise timing [some guidance is given ADA guidelines (American Diabetes Association, 2018b)]. This is a very important consideration that is beyond the intended aim of this review but studies addressing the effect of exercise on the prevalence and inter-individual variability of hypoglycemia in T2DM are lacking.

Besides anti-hyperglycemia drugs, lipid-lowering HMG-CoA reductase inhibitors (e.g., statins) are commonplace in the management of (pre)diabetes. In the 1990/2000s, it was shown that statin use may lead to mitochondrial dysfunction and muscle damage (Thompson et al., 1997; Päivä et al., 2005; Draeger et al., 2006; Schick et al., 2007), prompting a hypothesis that statin treatment may blunt the beneficial effect of exercise. Meex et al. (2010) found that statin treatment (between 5 and 40 mg/day of atorvastatin, simvastatin, rosuvastatin, or pravastatin) combined with regular aerobic plus resistance training more robustly increased insulin sensitivity than training alone. They observed equal weight loss and improved fitness and muscle mitochondrial function between groups but no improvements in fasting glucose or HbA1c (Meex et al., 2010). While this work showed that statins unlikely impair exercise adaptations to glucose metabolism and mitochondrial function, with different dosing regimens and the inclusion of individuals with/without diabetes treated with a mix of hyperglycemia-lowering drugs (metformin and/or sulfonylureas), is it difficult to ascertain the precise exercise-statin interaction from this work. In 2013, two studies examined exercise-statin interactions. Larsen and colleagues compared ten simvastatin-treated (10–40 mg/day for ∼5-years) hypercholesterolemic patients with untreated controls matched by age, weight, body mass index, fat percentage and VO_2_max. They found lower insulin sensitivity, lower muscle mitochondrial function, and higher HbA1c levels in statin-treated patients (Larsen et al., 2013). Meanwhile, Mikus et al. (2013) randomized thirty-seven obese individuals with metabolic syndrome to 12-weeks of aerobic exercise with or without simvastatin treatment (40 mg/day). They found that exercise-induced improvements in VO_2_max and muscle mitochondrial content were absent in the statin-treated group. Again, these studies indicate potential for statins to influence exercise-related factors but they do not allow one to conclude whether statin use directly influences changes in blood glucose control following exercise.

Other retrospective studies have also examined exercise-drug interactions. For example, in 2014, a small study (*N* = 14) by my group found that the increase in GLP-1- or arginine-stimulated insulin secretion following a single exercise bout was absent in T2DM patients who were drug-treated (Knudsen et al., 2015). This is similar to the above-described findings from Erickson et al. (2017b) who found blunted acute exercise-induced improvements in postprandial glucose in patients treated with multiple anti-hyperglycemics. However, Knudsen et al. (2015) did not examine specific drugs and was not designed to prospectively examine exercise-drug interactions. In their retrospective analysis of outcomes from a 12-week training study in patients with T2DM, Terada et al. (2013a) demonstrated that greater reductions in blood glucose were found when diabetic medications were taken less than 6-h prior to exercise compared to more than 6-h. These observations suggest that drug-exercise timing is important; however, outcomes were not derived from controlled drug administration but from retrospective analyses of patients’ drug diaries.

Unfortunately, published work that documents exercise-drug interactions vary in their study designs and target populations, and have mixed outcomes. Consequently, such work has not informed current clinical guidelines. With the exception of a brief comment on insulin-activity timing (American Diabetes Association, 2018c), as of 2018 no information regarding exercise-drug timing is currently provided in the ADA guidelines for preventing and treating T2DM. Although there is an urgent need for a larg-scale prospective trial specifically examining the interactions between anti-hyperglycemic medications and exercise to optimize blood glucose control for patients with T2DM, based on the equivocal evidence available it seems likely that bespoke and carefully monitored exercise-drug timing and dosing is required for patients on an individual basis.

### F. Weight Loss

The independent effects of exercise and weight loss on blood glucose control in (pre)diabetes have been well studied, showing that either approach may improve blood glucose control or insulin sensitivity (Goodpaster et al., 2003; Solomon et al., 2008, 2009; Dubé et al., 2011). However, their interactive effects are less understood. Furthermore, a lack of energy balance and prevention of consequent weight loss confounds the interpretation of many exercise studies. For example, we found that interval walking training improved blood glucose control in T2DM patients whereas continuous walking did not; however, interval walkers also displayed greater reduction in body fat mass, precluding a firm conclusion that the benefit was induced by interval walking *per se* (Karstoft et al., 2013).

Although data from Goodpaster et al. (2003) demonstrated the additive effects of diet-induced weight loss (10% of body mass via a 500–1000 kcal/day deficit) and exercise training (40 min at 75% HRmax, 4–6 times/week) on insulin sensitivity, their work did not reveal additive effects on blood glucose control (fasting glucose, or 2-h OGTT glucose). Our work also previously found that improvements in fasting glucose, 2-h OGTT glucose, or insulin sensitivity were equal in obese individuals with prediabetes randomized to 12-weeks of exercise training (60 min/day at 65% VO_2_max, 5 days/wk) either with or without 500 kcal/day deficit-induced weight loss (Solomon et al., 2008, 2009). A following study from Goodpaster et al. (2010) found that delaying initiation of physical activity during a weight loss intervention in severely obese individuals had no influence on metabolic outcomes, including improved fasting glucose at 6-months. That said, other studies have found that exercise is critical for maintaining improved glucose control. For example, Thomas and colleagues carefully examined the effects of weight regain on cardiometabolic risk factors following 6-months weight loss (10% of body weight via deficit of 600 kcal/day) with supervised exercise (walking at 60% of VO_2_max, 400 kcal/session, 5 days/week) in 100 metabolic syndrome patients (Thomas et al., 2010). They found that individuals randomized to continue exercise during controlled weight regain following initial weight loss, maintained improved blood glucose control while most metabolic variables deteriorated in those who ceased exercise (Thomas et al., 2010). Bouchonville et al. (2014) reported that fasting glucose, and glucose AUC and insulin sensitivity measured during OGTT were more robustly improved following 12-months of diet-induced weight loss (10% of body weight) plus exercise (90-min of combined aerobic and resistance, thrice weekly) when compared to either weight loss or exercise alone, in 100 obese individuals. The additional benefit conveyed by exercise plus diet-induced weight loss is supported by other recent work (Weiss et al., 2015; Francois et al., 2018). But the importance of exercise alone is also underpinned by outcomes from the IDES trial: Balducci et al. (2012) found that the magnitude of increase in fitness following exercise training (twice weekly supervised aerobic and resistance training plus exercise counseling) predicts improvement of cardiometabolic risk factors including HbA1c, independent of weight loss.

We currently lack precise information regarding the interaction between exercise and weight loss from large-scale randomized controlled trials in order to update clinical guidelines. Nonetheless, the above-described observations underpin the necessity of regular exercise to maintain and/or maximize the benefits of weight loss on blood glucose control in individuals with (pre)diabetes. Current clinical guidelines do not convey this sentiment.

### G. Inactivity/Sitting Time

From the early 2000s, data has emerged that physical inactivity (daily time spent being sedentary, i.e., sitting or lying while awake) is strongly associated with T2DM risk and interacts with the level of physical activity (Dunstan et al., 2004; Wilmot et al., 2012). This provides evidence that the amount of daily inactivity may influence the inter-individual variability in changes in blood glucose control following exercise training. Further evidence has also emerged demonstrating that interrupting sitting time can be a useful intervention for preventing and managing blood glucose control (reviewed in Dempsey et al., 2016). Additionally, in the 45 and up study published in van der Ploeg et al. (2012), association analyses showed that patients with T2DM must increase their physical activity level and reduce their sedentary time in order to reduce mortality. Such evidence has prompted the ADA to include a statement in their standards of medical care stating that, in addition to increased physical activity and regular exercise, individuals should reduce their sedentary time by breaking up prolonged bouts of sitting with light activity for a few minutes at least every 30 min (**Table 2**) (American Diabetes Association, 2018b). However, since large scale randomized controlled trials examining such phenomena in patients with T2DM are lacking, this guideline is not wholly evidence-based. Furthermore, it is not known when an interruption to sitting time would be best initiated, i.e., in the postabsorptive or postprandial state (see *Exercise-Meal Timing*). Additionally, training studies in diabetes patients seldom report objectively measured physical activity/inactivity levels; consequently, the influence of inactivity on the variability in glycemic outcomes following training is unknown. With advances in tri-axial accelerometry, methods for objectively quantifying sitting time and the transition to standing and activity can be easily implemented with increasing accuracy and low cost. As new data emerges over the coming years, clinical guidelines related to the interruption of sedentary time will be further optimized. In relation to this, since an exercise bout may influence total daily activity levels (Thompson et al., 2014), prospective trials are required to understand the impact of this on glucose control in diabetes.

### H. (Epi)genetics

Genome wide association studies have identified several genes associated with increased risk of developing T2DM (reviewed in Prasad and Groop, 2015). Some of these genes are also associated with glycemic outcomes from weight loss lifestyle interventions, such as the Diabetes Prevention Program and the Diabetes Prevention Study (reviewed in Weyrich et al., 2007). With specific reference to the effect of exercise training on blood glucose control, far fewer genetic studies have been published. As described earlier in this paper, the HERITAGE family study demonstrated large heterogeneity in glycemic outcomes following a 20-week exercise training intervention, where approximately 40% of the participants showed no change or an adverse direction of change in IVGTT-derived parameters of blood glucose control (Boulé et al., 2005). Findings from the HERITAGE family study have shown that leptin and leptin receptor gene polymorphisms and a leptin gene trait locus on 7q31 are associated with training-induced changes in the insulin response to IVGTT and fasting insulin, respectively (Lakka et al., 2003, 2004). Rate of glucose disappearance, insulin sensitivity, and disposition index during IVGTT are also improved more following training in C allele carriers at rs2180062 in the *FHL1* gene than in the T allele carriers (Teran-Garcia et al., 2007). Although the HERITAGE family study did not examine individuals with prediabetes or T2DM, in 2010 the study investigators examined whether 8 T2DM susceptibility variants (single nucleotide polymorphisms, SNPs, previously identified through genome-wide linkage analyses) could modulate changes in IVGTT-derived measures of glycemic control following 20 weeks of regular exercise training (Ruchat et al., 2010). After adjustment for multiple comparisons and adjusting for weight loss (change in waist circumference), the authors identified that a Pro12Ala SNP in the *PPAR*γ gene accounted for statistically significant variance in exercise-induced changes in the glucose disappearance rate (ΔK_g_, 2.81% of variance explained) glucose effectiveness (ΔS_g_, 1.83%), the acute insulin secretory response to glucose (ΔAIR_g_, 0.94%), and the disposition index (ΔDI, 2.15%) (Ruchat et al., 2010). The authors also found that carriers of the Ala allele had greater exercise-induced improvements in these IVGTT-derived variables (Ruchat et al., 2010). The findings from this work advanced our knowledge; however, only 8 SNPs were selected and HbA1c or 2-h OGTT glucose were not measured. Furthermore, since the publication date of that study in 2010, several more SNPs associated with diabetes risk have been identified. As such, there is a great need for similar studies in individuals with prediabetes or T2DM. That said, in Klimentidis et al. (2014) examined the influence of 65 T2DM-associated SNPs on the relationship between physical activity level and genetic risk score for T2DM. They found that the protective effect of physical activity was weakest among individuals with high genetic risk for T2DM. Their findings suggest that the role of physical activity in the prevention of diabetes may be blunted in those with high susceptibility for the disease. However, the causality of such correlative findings must be confirmed.

Besides genetics, epigenetics have received little attention in the context of the exercise and blood glucose control in pre(diabetes). Barrès et al. (2012) measured whole genome methylation as well as the methylation status of exercise responsive genes (PGC-1α, PDK4, and PPAR-δ) in skeletal muscle biopsies from healthy adults at rest and following a single exercise bout. Exercise induced a dose-dependent expression of PGC-1α, PDK4, and PPAR-δ, together with a marked hypomethylation on their respective promoters. The authors further showed that acute exercise caused a transient changes in the pattern of DNA methylation in adult skeletal muscle tissue (differentiated non-dividing somatic cells), and that DNA methylation was unaltered following 3-weeks of training despite increased RNA expression of PGC-1α and TFAM promoters (Barrès et al., 2012). This was a seminal observation in exercise biology since it demonstrated that DNA hypomethylation is a likely a transient mechanism involved in mRNA synthesis and that epigenetic regulation of the genome is dynamic to acute stimuli. However, whether promoter hypomethylation induces a functional influence on blood glucose control from exercise in individuals with (pre)diabetes, remains to be investigated.

In data published in 2015 from the HART-D study, a large-scale randomized controlled trial which determined the effect of 9-months of supervised exercise training on HbA1c in patients with T2DM, Stephens et al. (2015) measured the baseline skeletal muscle transcriptome before the intervention. The authors identified 186 genes with differential mRNA expressions between “responders” (training-induced decrease in HbA1c) and “non-responders” (no change in HbA1c) of which ∼25% of these differentially expressed genes were involved in substrate metabolism and mitochondrial dynamics (Stephens et al., 2015). Targeted qRT-PCR analyses of a selection of genes from their array demonstrated that the lack of training effect on HbA1c was linked to lower baseline expression levels of exercise-responsive genes. These included PPARα and ELOVL1, which play a role in lipid metabolism, and CHKB, CISD2, and FOXO1, which are involved in mitochondrial function. Such findings prove very useful in identifying molecular biomarkers of exercise effectiveness in T2DM, and individualized follow-up studies of the “non-responders” are needed in order to understand how their therapeutic benefit from exercise can be achieved.

From the data we have available in 2018, identified genetic/epigenetic/transcriptomic factors explain only a small amount of the variability in outcomes following training. For example, the HERITAGE family study found that less than 5% of the variance in glycemic outcomes following training was explained by 8 T2DM susceptibility variants (Ruchat et al., 2010). As -omics technologies improve and become more widespread and more accessible in exercise science, there is no doubt that metabolite, protein, and microRNA signatures, as well as DNA methylation loci, which predict the magnitude of the therapeutic effect of exercise on blood glucose control in individuals with (pre)diabetes will be identified. Evidence to support this notion was presented by Rowlands et al. (2014) who found multiple alterations in the transcriptome, the methylome, and microRNA arrays following 16-weeks of resistance or endurance training in obese Polynesian individuals with T2DM. Due to a lack of evidence, as of 2018 the ADA guidelines do not include any information on molecular biomarkers which may be used to inform exercise prescription. This is very likely to change over the next 10-years, particularly as outcomes from studies like the Molecular Transducers of Physical Activity Consortium (MoTrPAC) evolve.

### I. Direct Effect of Hyperglycemia and Poor Beta-Cell Function

Chronic exposure to high glucose levels deteriorates cellular function and/or causes apoptosis in tissues that regulate blood glucose control. For example, several groups including my own have found that *in vitro* exposure of differentiated skeletal muscle cells (myotubes) to prolonged (>24-h) hyperglycemia reduces insulin-stimulated glucose uptake (Aas et al., 2011; Green et al., 2012). Solomon et al. (2012) we confirmed these observations in humans showing that elevation of plasma glucose 5 mM above basal for 24-h reduced insulin sensitivity in healthy volunteers. Furthermore, primary myotubes isolated from hyperglycemic donors exhibit blunted muscle cell adaptations to electrical pulse stimulated contractions (Feng et al., 2015). Therefore, *in vitro* observations prompt one to hypothesize that chronic exposure to high blood glucose levels (the phenotype of T2DM) may blunt beneficial exercise adaptations. To test this hypothesis, in 2013 we examined the relationship between pre-intervention blood glucose control (HbA1c, fasting glucose, and 2-h OGTT glucose) and changes in glucose control following 3–4-months of exercise training (∼4–5 days/week, up to 60 min/session at 60–70% HRmax) in 105 individuals with prediabetes or T2DM. Interestingly, we found a U-shaped relationship suggesting that individuals with relativity well controlled hyperglycemia respond well to training while patients with poor blood glucose control have poor improvements or even a deterioration in blood glucose control following training (Solomon et al., 2013a). Another study found that fasting hyperglycemia was also associated with blunted improvements in 2-h OGTT glucose following 3-months of aerobic training in older obese individuals (Malin and Kirwan, 2012). Furthermore, the STRRIDE study found an inverse correlation between baseline fasting glucose and aerobic training-induced improvement in insulin sensitivity (S_i_ from IVGTT) in overweight individuals: S_i_ increased in participants with normal fasting glucose (<5.6 mM) but decreased in those with impaired fasting glucose (≥5.6 mM) (AbouAssi et al., 2015). However, these are correlational observations that do not imply causality and may be confounded by other influential variables. Additionally, some studies dispute a role for hyperglycemia in blunting the therapeutic action of exercise. Terada et al. (2013a) found that higher pre-exercise blood glucose concentrations were associated with greater decreases in blood glucose following a single exercise bout in patients with T2DM. Accordingly, van Dijk and colleagues demonstrated that greater HbA1c levels in T2DM patients correlated with greater decreases in mean glucose over the 24-h period following a single exercise bout (Van dijk et al., 2013). However, since the findings from Terada and van Dijk derive from single exercise bouts, they should not be extrapolated to reflect expected outcomes following chronic training.

To help understand the physiological mechanisms that potentially link hyperglycemia with exercise adaptations, the HERITAGE Family study demonstrated that pre-intervention glucose tolerance (K_g_ during IVGTT) influences training induced changes in glucose-stimulated insulin secretion (AIR_g_ during IVGTT) (Boulé et al., 2005). Boulé et al. (2005) found that in healthy non-diabetic subjects, training decreased AIR_g_ in individuals in the quartile with the highest K_g_ at baseline while AIR_g_ increased in those in the quartile with the lowest Kg. We repeated this work in people with prediabetes and T2DM, finding that exercise-induced improvements in blood glucose control were lowest in those with poorer pre-intervention pancreatic beta-cell function (Solomon et al., 2013b). This observation supports earlier work in patients with T2DM from Krotkiewski et al. (1985), and is complemented by a study conducted by Dela et al. (2004) who found that T2DM patients with a low C-peptide response to glucagon infusion had no improvement in glucose- or arginine-stimulated insulin secretion following 3-months of aerobic training. *In vitro* incubation of pancreatic beta-cell lines or primary islets in high glucose-containing medium reduces glucose-stimulated insulin secretory function (Donath et al., 1999; Maedler et al., 2002), findings we have also translated into human observations (Solomon et al., 2012). So it may be speculated that chronic exposure to high glucose levels may directly blunt otherwise beneficial exercise-mediated adaptations in the endocrine pancreas. In a pilot project to test that hypothesis, in 2013 we stratified T2DM patients with respect to their HbA1c value and examined insulin secretory function following a single exercise bout (Knudsen et al., 2015). We found that GLP-1 and arginine-mediated potentiation of glucose-stimulated insulin secretion was augmented by exercise in patients with well controlled glycemia (HbA1c < 6%) but worsened in patients with poor glucose control (HbA1c > 6%) (Knudsen et al., 2015). Although this was a small pilot study, it is the first evidence that causally links chronic exposure to hyperglycemia with blunted exercise adaptations in diabetes patients. In combination with above-described work (Krotkiewski et al., 1985; Dela et al., 2004; Solomon et al., 2013b), such data suggest that T2DM patients with poor beta-cell insulin secretory function may not optimally respond to exercise treatment modalities. Research studies are required to determine whether optimizing insulin secretory function in such patients prior to initiating training may restore beneficial exercise adaptations. Chronic cellular exposure to high glucose levels is typically linked with apoptosis driven by inflammation and/or oxidative stress, a process called glucotoxicity (Poitout and Robertson, 2008). However, it remains to be determined whether inflammatory or oxidative stress mechanisms underpin high glucose-induced prevention of beneficial exercise adaptations.

No study to date has examined whether exposure to experimental hyperglycemia (via infusion) or rapid normalization of hyperglycemia in diabetes patients (via insulin or sodium-glucose cotransport inhibitors drugs) can influence exercise adaptations. It remains to be investigated whether glucotoxicity directly influences exercise training-induced improvements in blood glucose control. Knowledge gained from answering such a research question would further inform ADA guidelines and therefore enable clinicians to enhance the management of their patients’ hyperglycemia. Such knowledge would also help individualize lifestyle intervention approaches if indeed glucose lowering and/or beta-cell optimizing therapy is required in some patients prior to initiating an exercise regime.

Since several other descriptive characteristics such as age, sex, race, body weight, or duration of diabetes (years since diagnosis) or specific dietary nutrients, may independently influence the above described contributing factors, one may speculate that these may influence exercise-mediated effects on blood glucose control in patients with (pre)diabetes. The same may also be true of activity compensation where an exercise bout may negatively influence total daily activity levels (Thompson et al., 2014). Family history of diabetes should also be considered in future work since individuals with a diabetic parent exhibit a blunted post-exercise insulin-mediated glycogen storage response (Price et al., 1996). However, following regression analyses in one of my own studies (Solomon et al., 2013b), neither age, BMI, sex, or time since diabetes diagnosis had any influence on the hyperglycemia-lowering effect of exercise training. Regression analyses from van Dijk and colleagues also support that neither age, BMI, diabetes duration, or drug treated influence exercise-induced blood glucose control in patients with T2DM (Van dijk et al., 2013). That said, no study has prospectively examined the role of such variables on blood glucose control following training in individuals with prediabetes or T2DM. One exception may be the Look-AHEAD study, a randomized controlled trial examining the effect of an intensive lifestyle intervention (combined diet and exercise induced weight loss) on T2DM remission in 5145 patients. The investigators found that longer-term remission (after 2 to 4-years follow-up) was more likely in patients not using insulin with less than 2-year duration of diabetes, a lower baseline HbA1c, and a greater first-year weight loss (Gregg et al., 2012). Although Look-AHEAD was not an exercise training study *per se*, it does indeed highlight factors to be considered in future exercise studies.

In the free-living “real world” setting, all of the above-described contributing factors play a role in the notable heterogeneity in the therapeutic blood glucose lowering response to exercise in people with (pre)diabetes. In the lab setting, where exercise is supervised and standardized, the influence of several of these above-described sources of variability, particularly exercise adherence, can be controlled and therefore minimized. Yet, in the free-living “real world” setting there are behavioral (desire, self-image, motivation) and environmental (climate, weather, terrain) barriers combined with abundant access to activity reducing transport modalities (cars, busses, trains, elevators, escalators, conveyer belts) which influence the adherence to exercise and thereby encourage an inactive lifestyle. Thus, the true challenge to maximizing the therapeutic potential of exercise is immense.

## Where Do We Go From Here?

The purpose of this review was to examine inter-individual variability in the blood glucose lowering effect of exercise in individuals with T2DM, and to identify the sources of such variability. Interpretations should not be extrapolated to other variables (e.g., lipids, blood pressure, etc.), nor should a non-response in blood glucose control following exercise be considered to convey a non-response in other variables. Due to a lack of standardization of study design, differences in methods/assays, variations in timing of post-training measurements, heterogeneity of subject demographics between trials, and probably most importantly, a lack of measurement of clinical diagnostic measures for assessing blood glucose control, a systematic review and meta-analysis on this topic is not possible. However, from the evidence presented above it is highly likely that inter-individual variability in the changes in blood glucose control following exercise exists in the context of T2DM and that true non-responders will be identified. In doing so, one must be aware that “non-responder” does not mean “never responder.” Identifying an adverse outcome to a particular intervention should be embraced as a challenge to overcome. By doing so, the knowledge gained will ultimately maximize the therapeutic benefits of exercise for all patients.

**FIGURE 3:**
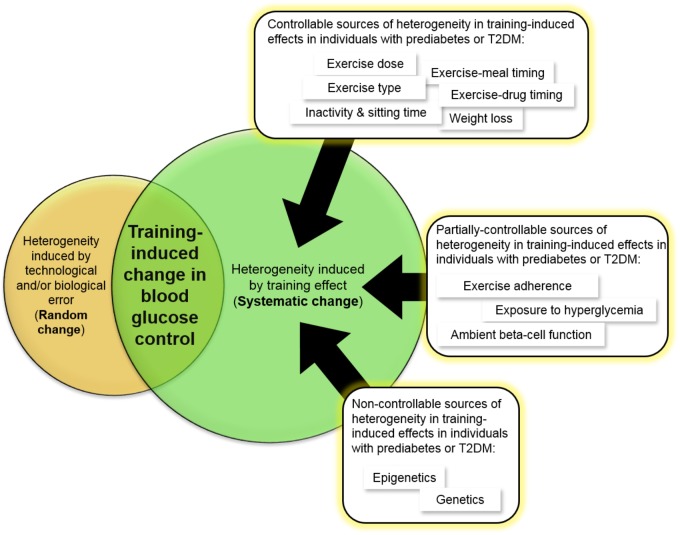
Evidence-based sources of inter-individual variability in the blood glucose lowering effects of exercise in individuals with prediabetes or T2DM. Other sources that have not been adequately studied to conclusively state that they contribute to this variability in individuals with prediabetes or T2DM include age, sex, race, body weight, family history of diabetes, and duration of diabetes.

Going forward, several sources of variability have been identified (**Figure 3**), and I propose that exercise dose (including frequency, intensity, and time above habitual activity level), exercise type, exercise adherence, exercise-meal timing, exercise-drug timing, and drug name and dosing, and objectively measured physical activity level and sedentary time, should always be considered in a study design and reported in publications. Among many published studies, I admit that I too have been guilty of not always including such details in my papers, either through accidental omission or failure to record such data. Remedying this in future will increase the quality of work in the field and enable comparisons between independent studies. This would facilitate the accurate calculation of technical error of measurement and eventually establish an evidence-based “reference range” indicative of a clinically meaningful exercise-induced improvement in blood glucose control. Such an approach would then enhance the reliability of information used to inform clinical guidelines. That said, **Table 3** highlights the current science gaps that must be urgently filled if we are to understand how to maximize the therapeutic benefit of exercise on blood glucose control for all individuals with prediabetes or T2DM. The new knowledge that will emerge in the next 5–10 years will couple genetic, transcriptomic, epigenetic, and physiological factors with knowledge of exercise dosing, exercise-meal timing, and exercise-drug interactions to help maximize the therapeutic benefit of exercise for all individuals, including those at risk of developing diabetes or those already with T2DM. This creates great confidence that we will soon successfully control the incidence of this preventable disease.

**Table 3:** Science gaps which, if filled, will increase our understanding of inter-individual variability in the therapeutic blood glucose lowering effect of exercise for individuals with prediabetes and/or type 2 diabetes.

Science gaps	
1	A randomized controlled trial of exercise training to determine the patient-by-treatment interaction for the change in blood glucose control (HbA1c, fasting glucose, and 2-h OGTT glucose) is needed in people with prediabetes and T2DM. This would help accurately quantify inter-individual variability and identify true non-responders.
2	A study to determine the inter-individual variability in blood glucose control caused by different exercise doses (frequency, intensity, and time) is needed in individuals with prediabetes or T2DM.
3	A study to determine the inter-individual variability in blood glucose control caused by different types of exercise is needed in individuals with prediabetes or T2DM.
4	A description of psychological barriers, behavioral barriers, and environmental barriers to implementing lifestyle changes and incorporating exercise into diabetes treatment should be included in clinical guidelines.
5	A study to determine the optimal exercise-meal timing needed to maximize postprandial glucose control in individuals with prediabetes or T2DM is required.
6	There is an urgent need for a large-scale prospective trial specifically examining the interactions between exercise and anti-hyperglycemic medications to optimize blood glucose control for patients with T2DM.
7	A large scale randomized controlled trial examining the interruption of sitting time with light activity (and its pre-postprandial timing) in patients with T2DM is needed.
8	There is a need for studies to identify metabolite, protein, or microRNA signatures, as well as DNA methylation loci, which predict the magnitude of the therapeutic effect of exercise on blood glucose control in individuals with prediabetes of T2DM.
9	A study determining whether exposure to experimental hyperglycemia (via infusion) or rapid normalization of hyperglycemia in diabetes patients (via insulin or sodium-glucose cotransport inhibitors drugs) can directly influence exercise adaptations is needed.
10	Exercise dose (including frequency, intensity, and time above habitual activity levels), exercise type, exercise adherence, exercise-meal timing, exercise-drug timing, and drug name and dosing, and objectively measured physical activity levels and sedentary time, should always be considered in a study design and be reported in publications.


*These future approaches will optimize clinical exercise guidelines, and ultimately help maximize the therapeutic blood glucose lowering effects for all patients. Further detail and references are in the main text.*

## Author Contributions

TS wrote the manuscript and takes responsibility for the integrity of its content.

## Conflict of Interest Statement

The author declares that the research was conducted in the absence of any commercial or financial relationships that could be construed as a potential conflict of interest.
